# Forecasting the development of antimicrobial resistance of *S. aureus*

**DOI:** 10.3389/froh.2024.1514070

**Published:** 2025-01-09

**Authors:** Oleh Shemetov, Mariia Faustova, Tetiana Perepelova, Hennadii Balia, Ihor Pavlish, Galina Loban'

**Affiliations:** ^1^ Department of Prosthetics Dentistry with Implantology, Poltava State Medical University, Poltava, Ukraine; ^2^ Department of Microbiology, Virology and Immunology, Poltava State Medical University, Poltava, Ukraine

**Keywords:** antimicrobial resistance, *S. aureus*, antibiotics, odontogenic infections, phlegmons, forecasting

## Abstract

Today, about 15.0% of odontogenic pathology is caused by *Staphylococcus aureus* (*S. aureus*). The aim of the study was to predict the development of antimicrobial resistance of *S. aureus* based on retrospective data.

**Methods:**

A total of 425 patients undergoing treatment for odontogenic infectious diseases of the facial area during 2019–2023 were involved in the study. The object of the study was 106 clinical isolates of *S. aureus* that were isolated and identified from patients. Determining the sensitivity of the obtained isolates to antimicrobial drugs was carried out using Vitek antimicrobial susceptibility testing (Biomerioux, France) and analyzed according to the breackpoint tables of the EUCAST. Prediction of the development of antimicrobial resistance of *S. aureus* to various antibiotics was carried out on the basis of the received sensitivity data of the studied isolates in 2019–2023 using the exponential smoothing method.

**Results:**

The antimicrobial resistance of *S. aureus* isolates to various antibiotics changed annually during 2019–2023. The level of resistance of *S. aureus* isolates to benzylpenicillin wavered between 40%–50% from 2019 to 2023 with the trend of an 18.0% increase over the next five years. A uniform plateau of antimicrobial resistance of *S. aureus* to cefoxitin is predicted at the level of 32.0% during 2024–2028. We recorded the highest portions of *S. aureus* resistant to norfloxacin (33.3%) and ciprofloxacin (16.7%) in 2023 with prediction of its increasing in the next five years within the range of 20.0%. It was established that *S. aureus* may reach 100.0% resistance to gentamicin in 2027. According to exponential smoothing, the level of *S. aureus* resistance to amikacin will increase by 22.7% over the next five-year period. Moreover, representatives of this species of bacteria can develop complete (100.0%) resistance to tetracycline as early as 2027.

**Conclusions:**

Mathematical prediction of the development of antimicrobial sensitivity of *S. aureus* isolates showed a high probability of its development to antibiotics of all groups in the next five years.

## 1 Introduction

Almost a quarter of surgical pathology in the world is due to odontogenic infections (1, 2). Considering the fact that they develop during the spread of microorganisms through the destroyed tissues of the teeth into the underlying tissues, the bacterial etiological factor is obvious and indisputable (2, 3). The development of laboratory and microbiological methods of research contributes to the revision of the patterns of the microbiota of foci of odontogenic infection recently (4). Thus, more and more often there are scientific works indicating the role of gram-negative rods and spirochetes with a predominantly anaerobic type of respiration in the development of this pathology (5–8). However, along with this, the participation of gram-positive cocci, although somewhat reduced in recent years, continues to hold a leading position in odontogenic infections (9, 10).

Today, about 15.0% of odontogenic pathology is caused by *Staphylococcus aureus* (*S. aureus*) (2). It is worth noting that this microorganism is one of the so-called ESKAPE pathogens, which are characterized by high virulence and multiple resistance to various antimicrobial agents (11). According to literature data, the frequency of development of methicillin resistance among strains of *S. aureus* in the world varies, but in general it is not less than 20.0% for highly developed countries (12–14). Research in 2022 shows that the proportion of multi-resistant *S. aureus* in Ukraine exceeds 35.0%. Taking into account an ineffective state measures to counter the development of resistance and the war on the territory of Ukraine, it can be assumed that this indicator is not final and may increase in the following years (15, 16).

The aim of the study was to predict the development of antimicrobial resistance of *S. aureus* based on retrospective data.

## 2 Methods

### 2.1 Ethics

Written informed consent was obtained from each subject after a detailed explanation of the aim and protocol of the study, which was conducted in accordance with the ethical principles set forth in the Declaration of Helsinki for Ethical Principles for Medical Research Involving Human Subjects. The study was approved by the commission on biomedical ethics of the Poltava State Medical University (minutes #188 dated November 25, 2020).

### 2.2 Study population and specimens’ collection

A total of 425 patients undergoing treatment for odontogenic infectious diseases of the facial area (abscesses and phlegmons) during 2019–2023 were involved in the study.

The criteria for the inclusion of patients in the study was the confirmed diagnosis of odontogenic infectious diseases of the skin and subcutaneous tissue (abscesses and phlegmons) of the facial area, subject to consent to participate in the study. Exclusion criteria were non-compliance with the diagnosis, pregnancy, diabetes, presence of congenital or acquired immunodeficiency, mental disorders, taking antibiotics before collecting specimens, and refusal to participate in the study.

Along with the general clinical methods of research, specimens were collected from each patient for further microbiological research in order to isolate the etiologically significant pathogen and determine its sensitivity to antibiotics. Purulent exudate was collected with a sterile probe-tampon immediately after dissection of the focus of infection, followed by transfer to Amies transport nutrient medium. Cultivation of the material was carried out using a standard culture method on blood agar, meat-peptone agar and yolk-salt agar at a temperature of 37℃. The final identification of microorganisms was carried out with the automatic bacteriological analyzer Vitek compact (Biomerioux, France).

The object of the study was 106 clinical isolates of *S. aureus* that were isolated and identified from patients. Determining the sensitivity of the obtained isolates to antimicrobial drugs was carried out using Vitek antimicrobial susceptibility testing with AST-GP67 cards (Biomerioux, France) and analyzed according to the breackpoint tables of the EUCAST (current version for every year of study). The contents of AST-GP67 cards for Vitek 2 Systems is provided in Supplementary Table S1.

### 2.3 Statistical analysis

Mean, standard deviation, median, minimum, maximum value of frequency and percentage were used for descriptive statistics.

Prediction of the development of antimicrobial resistance of *S. aureus* to various antibiotics was carried out on the basis of the received sensitivity data of the studied isolates in 2019–2023 using the Holt's exponential smoothing (HES) method. This is a method of mathematical transformation for forecasting time series, in which each subsequent iteration takes into account all previous values of the series, but the degree of consideration decreases according to the exponential law (17). HES also called double Exponential Smoothing performs the forecasting of data with the trend formation not counting seasonality (18). It involves a forecast equation and two smoothing equations (one for the level and one for the trend). The standard exponential smoothing formulas (1–3) were used for mathematical processing of the results:

(1) $$\hat{Y_{t+hIt}}=l_{t}+hb_{t}$$

(2) $$l_{t}=\alpha y_{t}+(1-\alpha )(l_{t-1}+b_{t-1})$$

(3) $$b_{t}=\beta^{*}(l_{t}-l_{t-1})+(1-\beta^{*})b_{t-1},$$

where: *l_t_* - an estimate of the level of the series at time *t*; *b_t_*- an estimate of the trend of the series at time *t*; α - smoothing parameter for the level, 0 ≤ *α* ≤ 1; β* - smoothing parameter for the trend, 0 ≤ β* ≤ 1.

Mathematical analysis was carried out using the license package of the program StatPlus:macPro license program, AnalystSoft Inc. 2024 (USA).

## 3 Results

In general, the obtained results indicated the average similarity of the frequency of the development of antimicrobial resistance to different groups of antibiotics among isolates of *S. aureus* isolated from patients with odontogenic phlegmons and abscesses (Table 1). This provided the basis for further combined statistical processing of the results.

**Table 1:** The frequency of the development of antimicrobial resistance of *S. aureus* under conditions of odontogenic infections of the soft tissues of the facial area.

Antibiotics	Odontogenic abscesses (*n* = 37)		Odontogenic phlegmons (*n* = 69)	
	Abs.	%	Abs.	%
Benzylpenicillin	18	48.7	31	44.9
Cefoxitin	10	27.0	23	33.3
Methicillin (MRSA)	11	29.7	15	21.7
Norfloxacin	9	24.3	15	21.7
Ciprofloxacin	6	16.2	9	13.0
Amikacin	18	48.7	32	46.4
Gentamicin	21	56.8	38	55.1
Erythromycin	17	45.9	28	40.6
Tetracycline	16	43.3	29	42.0

As a result of a retrospective study of the sensitivity to antibiotics of pathogens that cause infectious and inflammatory diseases of the face, it was established that the resistance of *S. aureus* isolates to various antibiotics changed annually during 2019–2023.

**Figure 1:**
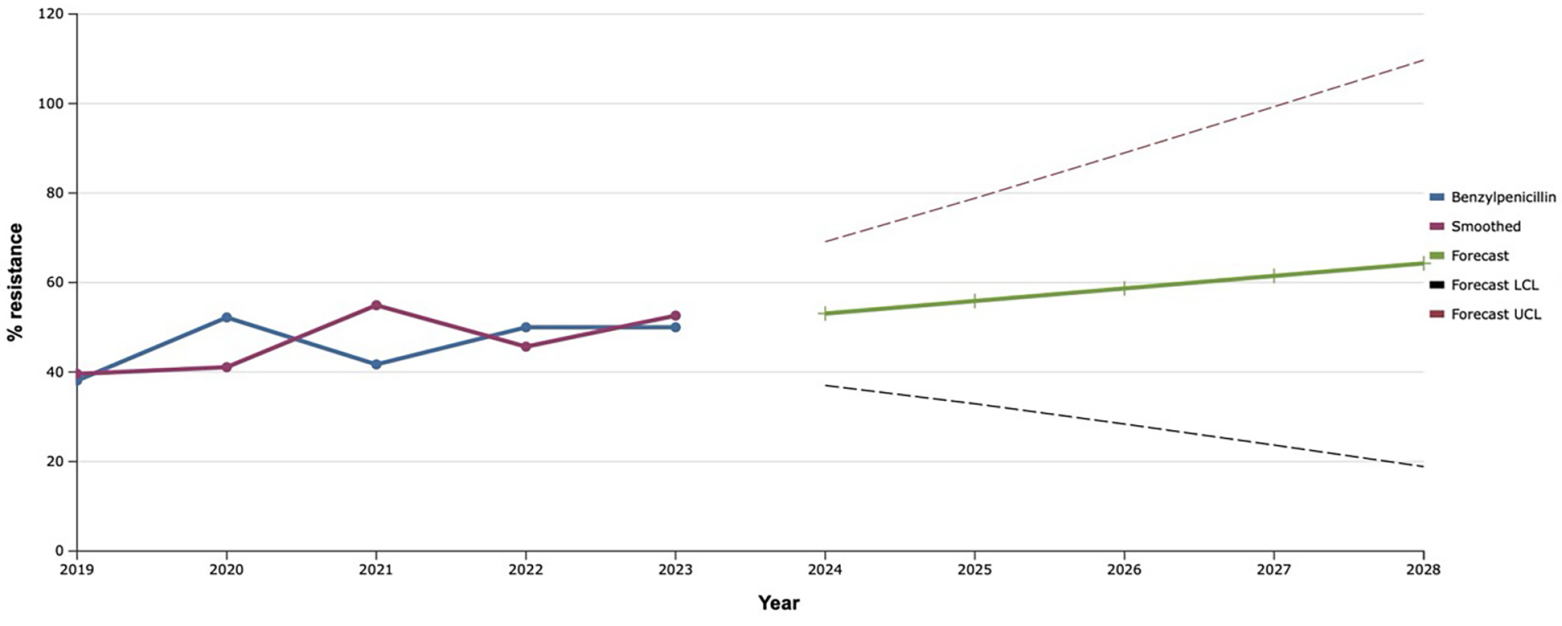
Forecasting the development of *S. aureus* resistance to benzylpenicillin based on retrospective data from 2019 to 2023 (screenshot of StatPlus: macPro license program, AnalystSoft Inc. 2024); Smoothed, the smoothed value at time; Forecast, the predicted value at time; Forecast LCL, forecast lower control limit; Forecast UCL, forecast upper control limit.

The level of resistance of *S. aureus* isolates to benzylpenicillin, by which resistance to all penicillins is assessed, wavered between 40%–50% from 2019 to 2023 (Figure 1). However, the exponential smoothing method clearly outlined the trend of an 18.0% increase in the proportion of *S. aureus* resistant to benzylpenicillin over the next five years. Thus, according to mathematical forecasting, the frequency of detection of antimicrobial resistance of *S. aureus* to benzylpenicillin, and hence to all penicillins, will reach 64.3% in 2028.

**Figure 2:**
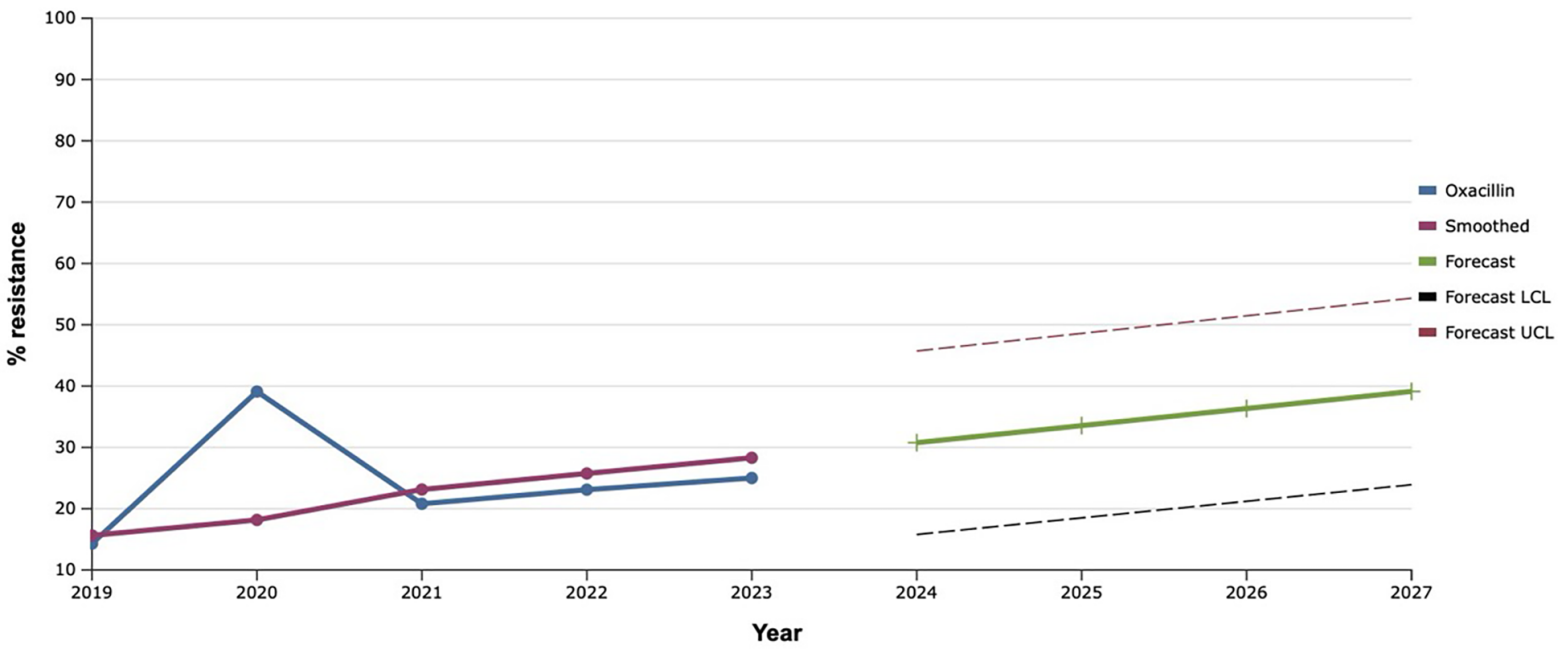
Forecasting the development of *S. aureus* resistance to oxacillin based on retrospective data from 2019 to 2023 (screenshot of StatPlus:macPro license program, AnalystSoft Inc. 2024); Smoothed, the smoothed value at time; Forecast, the predicted value at time; Forecast LCL, forecast lower control limit; Forecast UCL, forecast upper control limit.

Throughout the study period, the resistance of *S. aureus* isolates to oxacillin varied within 14%–39.0%. That is why, based on such retrospective data, it was mathematically established that the level of staphylococci resistant to this antibiotic over the next five years would increase by 17.0% compared to the level in 2023 (Figure 2).

**Figure 3:**
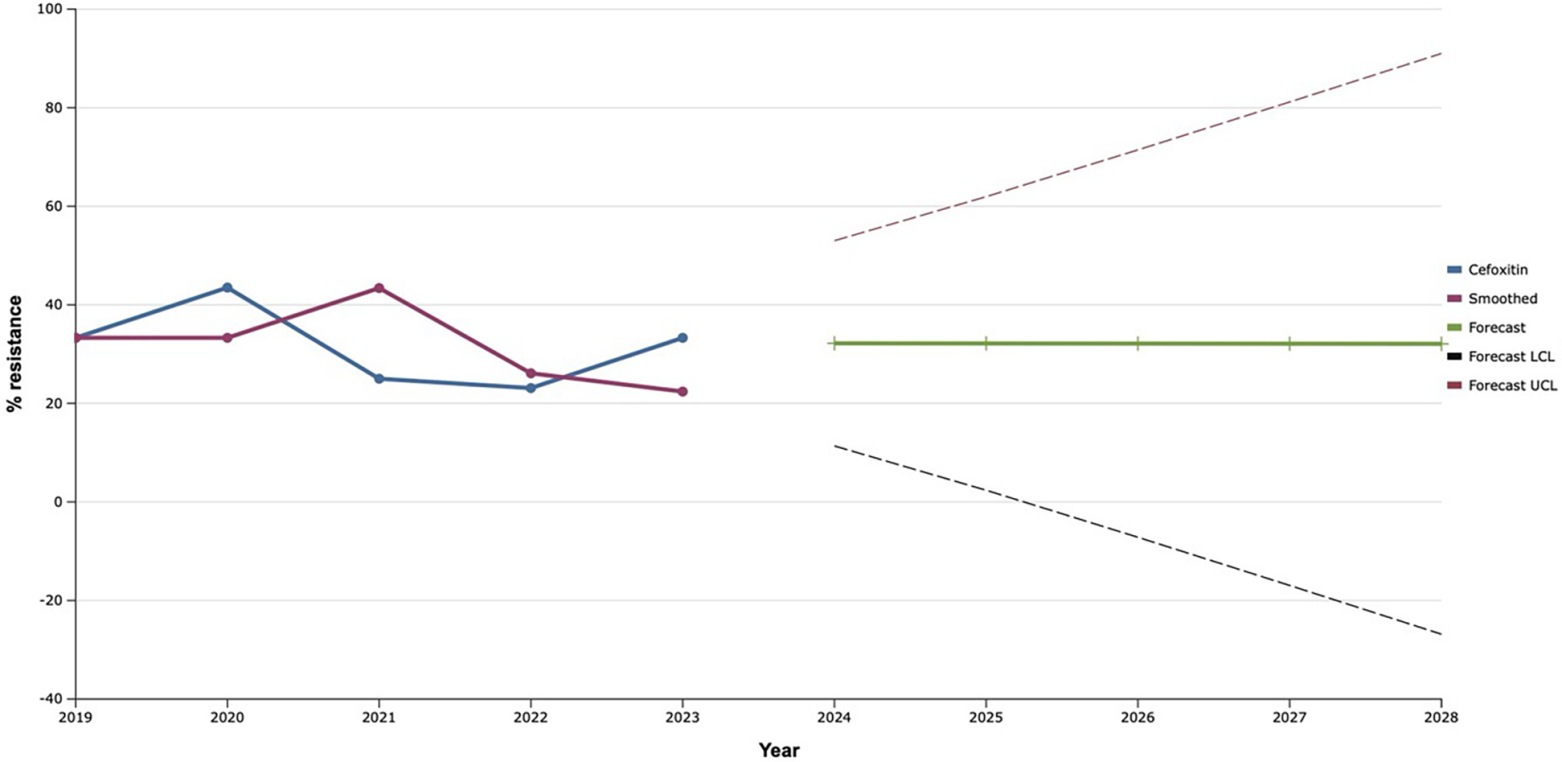
Forecasting the development of *S. aureus* resistance to cefoxitin based on retrospective data from 2019 to 2023 (screenshot of StatPlus: macPro license program, AnalystSoft Inc. 2024); Smoothed, the smoothed value at time; Forecast, the predicted value at time; Forecast LCL, forecast lower control limit; Forecast UCL, forecast upper control limit.

In the course of the study, a slight decrease in the level of resistance of investigated isolates to cefoxitin was observed in 2021–2022 to almost 20.0% with a 1.4-fold increase in the following year 2023 (Figure 3). Based on these data, a uniform plateau of antimicrobial resistance of *S. aureus* to cefoxitin is predicted at the level of 32.0% during 2024–2028.

**Figure 4:**
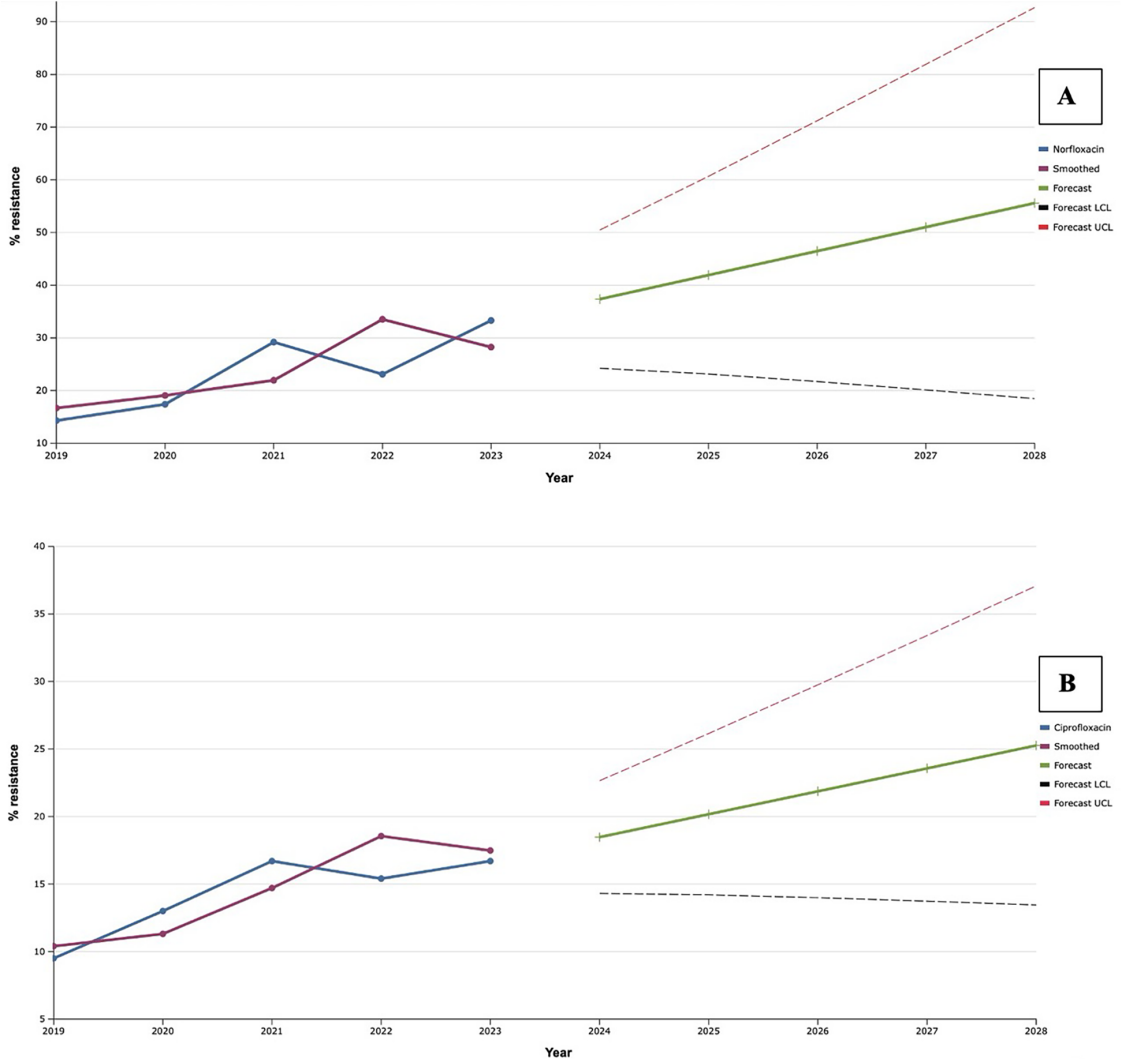
Forecasting the development of *S. aureus* resistance to norfloxacin **(A)** and ciprofloxacin **(B)** based on retrospective data from 2019 to 2023 (screenshot of the StatPlus:macPro license program, AnalystSoft Inc. 2024); Smoothed, the smoothed value at time; Forecast, the predicted value at time; Forecast LCL, forecast lower control limit; Forecast UCL, forecast upper control limit.

The level of antimicrobial resistance of clinical strains of *S. aureus*, isolated from patients with infectious inflammatory diseases of soft tissues of facial area, to fluoroquinolones gradually increased during 2019–2021 (Figure 4). Despite the slight decrease in this indicator in 2022, in the following year 2023, we recorded the highest portions of *S. aureus* resistant to norfloxacin (33.3%) and ciprofloxacin (16.7%). That is why, after the exponential smoothing analysis, a clear trend towards an increase in the level of resistance of *S. aureus* to fluoroquinolones was revealed. Thus, according to the results of the analysis, the proportions of ciprofloxacin-resistant isolates of this species will increase from 18.5% to 25.3% during 2024–2028. Norfloxacin showed a similar trend: the predicted proportion of *S. aureus* resistant to it increased steadily from 37.4% in 2024 to 55.6% in 2028. Taking into account the fact that the level of fluoroquinolone resistance is established based on the result of the sensitivity of staphylococci to norfloxacin, we can assume a predicted increase of the last one in the next five years within the range of 20.0%.

**Figure 5:**
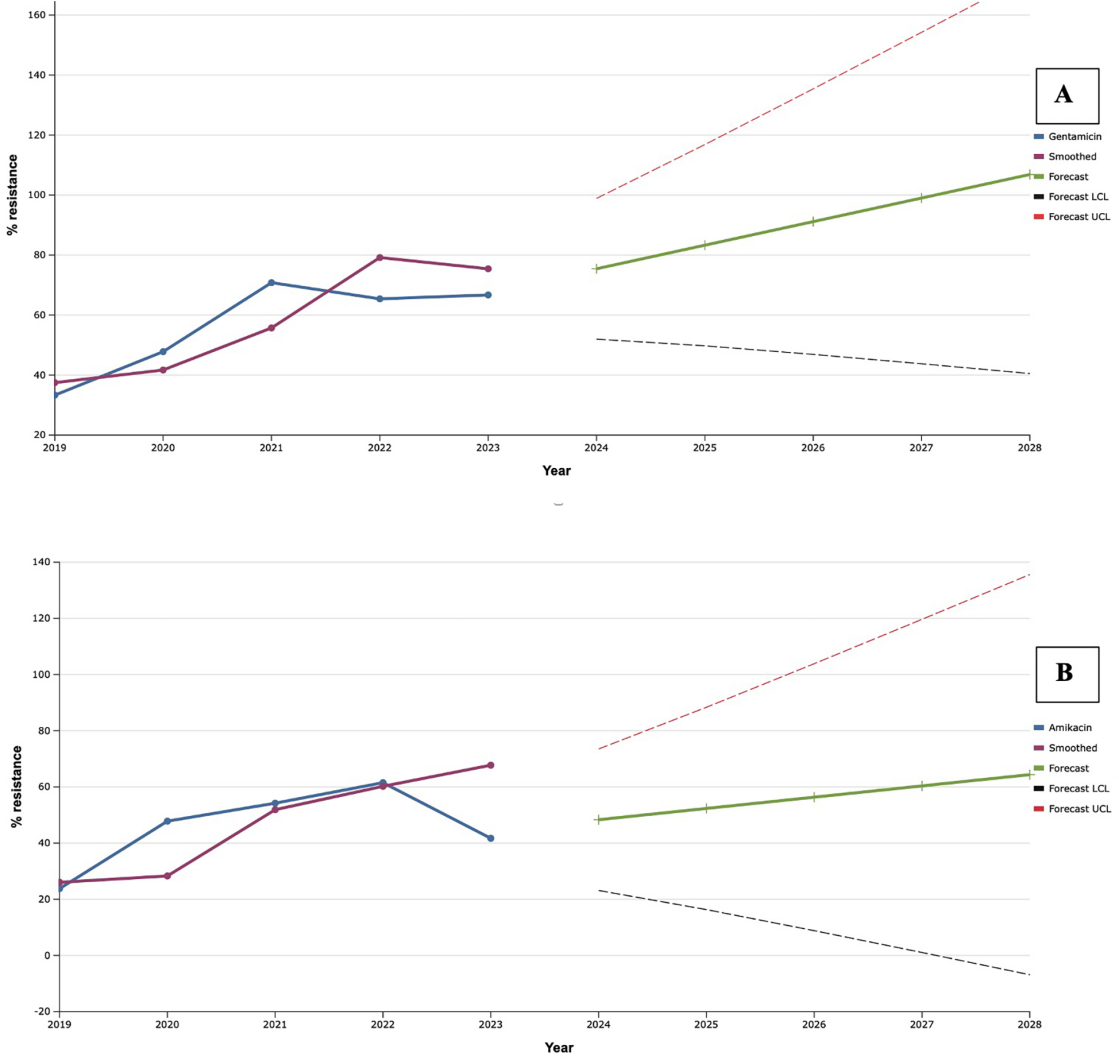
Forecasting the development of S. aureus resistance to gentamicin **(A)** and amikacin **(B)** based on retrospective data from 2019 to 2023 (screenshot of the StatPlus: macPro license program, AnalystSoft Inc. 2024); Smoothed, the smoothed value at time; Forecast, the predicted value at time; Forecast LCL, forecast lower control limit; Forecast UCL, forecast upper control limit.

As a result of the study, a rapid increase in the level of antimicrobial resistance of *S. aureus* to aminoglycosides was established during 2019–2021 (Figure 5), when record resistance to gentamicin was established (70.8%). Along with this, resistance to amikacin continued to increase and was maximal (61.5%) in 2022. Despite the fact that in 2022–2023, resistance to gentamicin decreased by 24.0% compared to the maximum indicator, mathematical forecasting indicated a potential increase in the level of resistance to this antibiotic among isolates of *S. aureus* in the next five years by 33.3%, compared to an indicator of 2023. At the same time, *S. aureus* may reach 100.0% resistance to gentamicin in 2027.

A 20.2% decrease in the level of resistance of the studied isolates to amikacin in 2023, compared to the previous year, contributed to the formation of a less rapid trend of the predicted results. Thus, according to exponential smoothing, the level of *S. aureus* resistance to amikacin will increase by 22.7% over the next five-year period (from 48.3% in 2024 to 64.4% in 2028).

**Figure 6:**
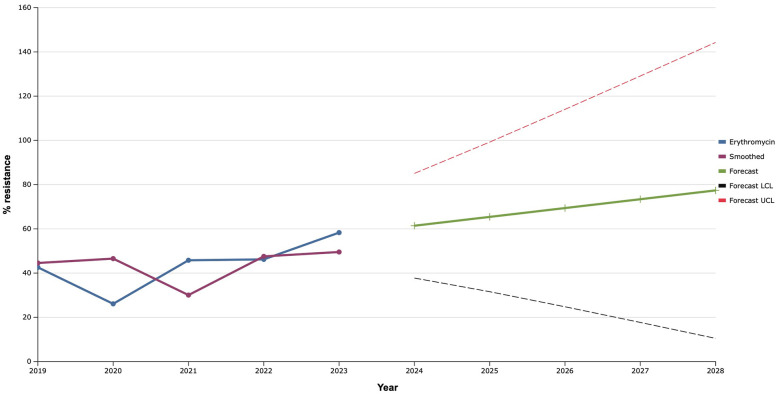
Forecasting the development of S. aureus resistance to erythromycin based on retrospective data from 2019 to 2023 (screenshot of StatPlus:macPro license program, AnalystSoft Inc. 2024); Smoothed, the smoothed value at time; Forecast, the predicted value at time; Forecast LCL, forecast lower control limit; Forecast UCL, forecast upper control limit.

During 2019–2022 erythromycin demonstrated mostly stable effectiveness against clinical isolates of *S. aureus* with a frequency of resistance at the level of 40.0%, with the exception of 2020, when a decrease in the proportion of resistant strains to 26.1% was noted (Figure 6). However, in 2023, the level of antimicrobial resistance of *S. aureus* isolates to erythromycin increased to almost 60.0%. Therefore, as a result of mathematical forecasting, a clear tendency to increase the frequency of isolation of *S. aureus* resistant to it was established in the future. Taking into account that resistance to erythromycin is evaluated for the presence of macrolide resistance, this forecast indicated a probable increase in the level of the it to 77.4% in 2028, what is 35.0% higher than the average indicator of resistance of *S. aureus* to macrolides in 2019–2023.

**Figure 7:**
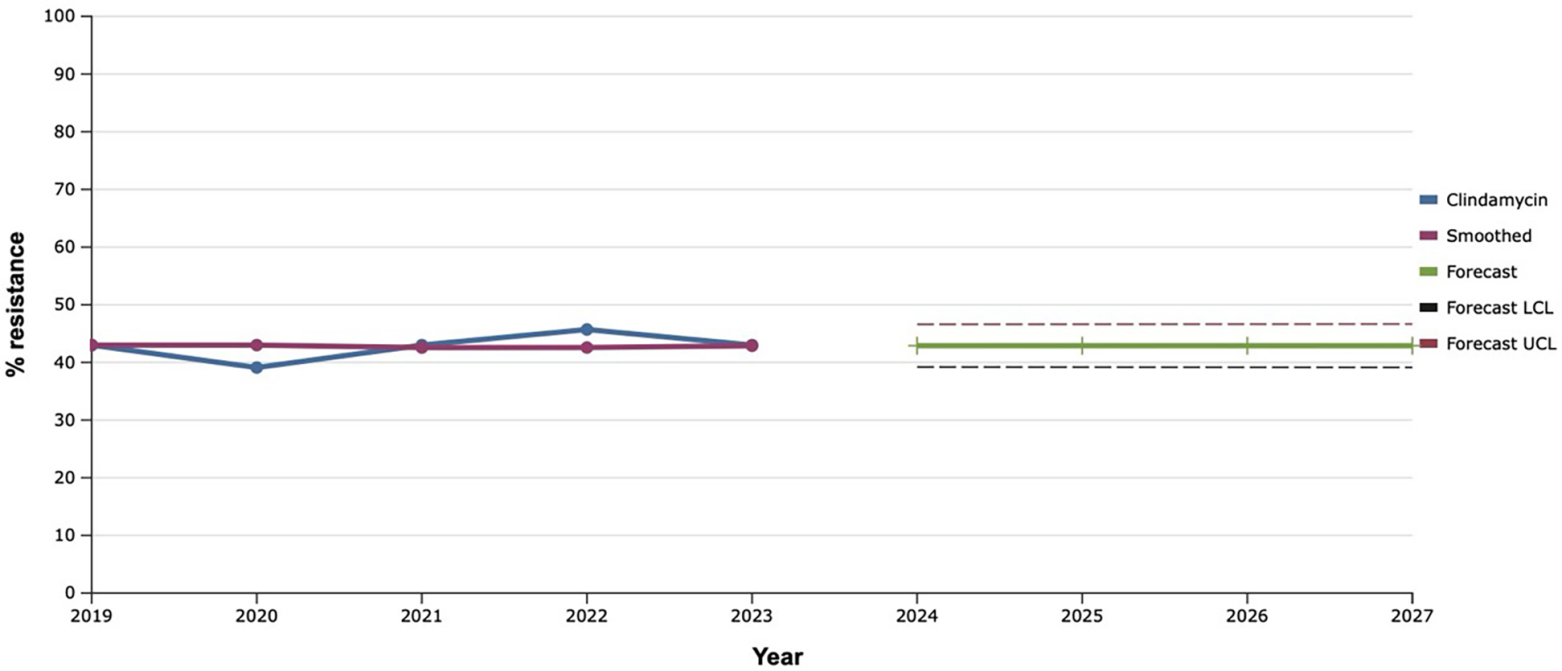
Forecasting the development of S. aureus resistance to clindamycin based on retrospective data from 2019 to 2023 (screenshot of StatPlus:macPro license program, AnalystSoft Inc. 2024); Smoothed, the smoothed value at time; Forecast, the predicted value at time; Forecast LCL, forecast lower control limit; Forecast UCL, forecast upper control limit.

Among lincosamides, the iinvestigated bacterial cultures were tested for clindamycin, the level of resistance to which almost did not change during 2019–2023. Therefore, the fact of the predicted plateau (around 43.0%) in the further development of S*. aureus* resistance to clindamycin over the next five years was natural (Figure 7).

**Figure 8:**
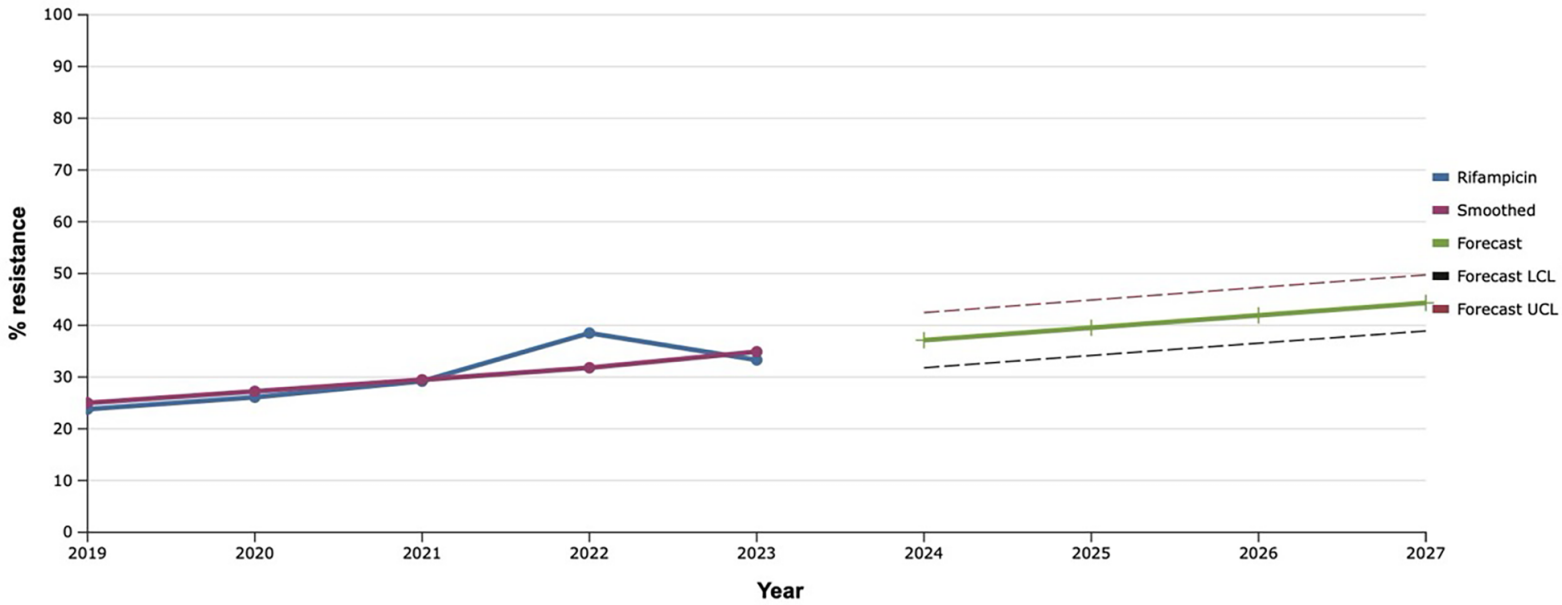
Forecasting the development of S. aureus resistance to rifampicin based on retrospective data from 2019 to 2023 (screenshot of StatPlus:macPro license program, AnalystSoft Inc. 2024); Smoothed, the smoothed value at time; Forecast, the predicted value at time; Forecast LCL, forecast lower control limit; Forecast UCL, forecast upper control limit.

The sensitivity of *S. aureus* isolates to rifampicin has been gradually increasing since 2019, reaching a maximum value of 38.5% in 2022. That is why, using the mathematical prediction method, it was established that the level of rifampicin-resistant *S. aureus* would increase to 46.7% in 2027 (Figure 8).

**Figure 9:**
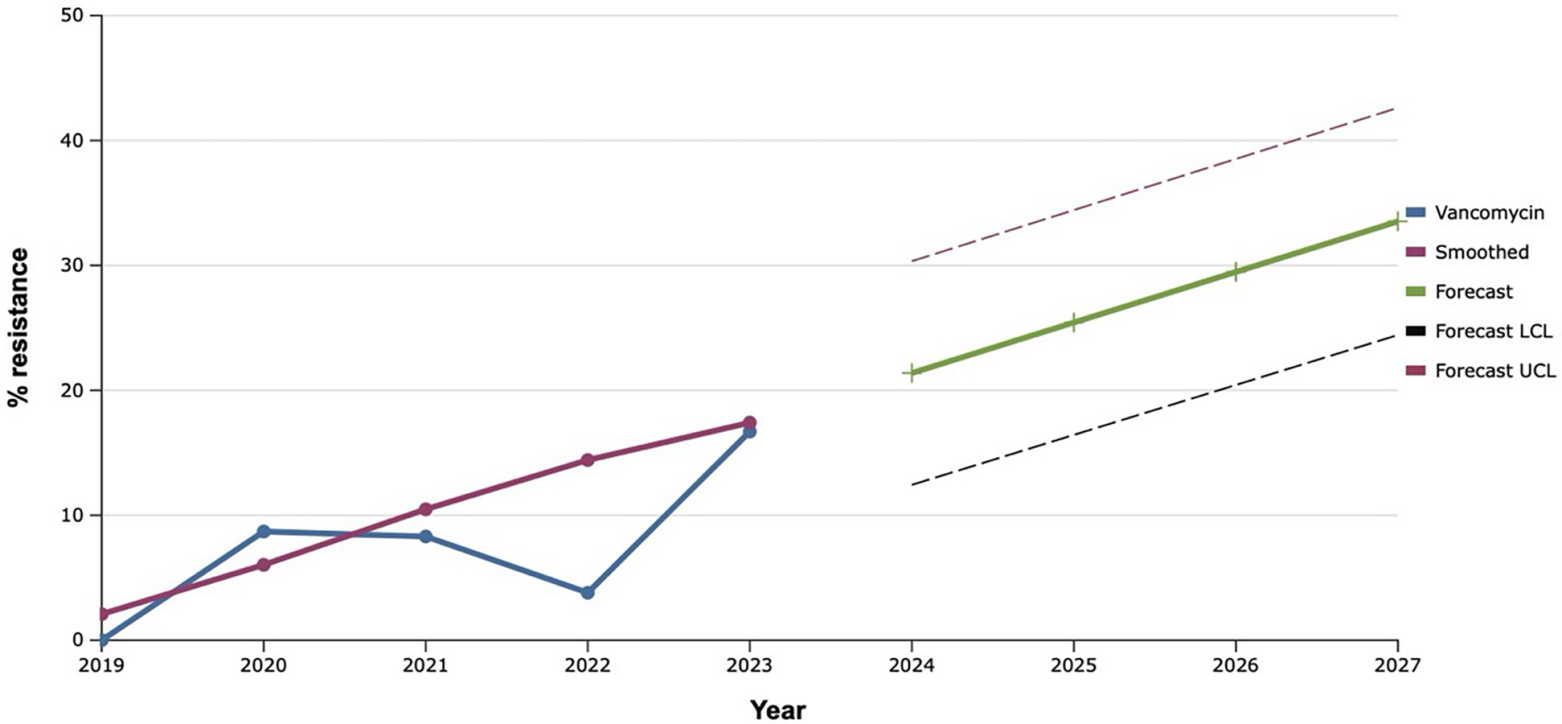
Forecasting the development of S. aureus resistance to vancomycin, based on retrospective data from 2019 to 2023 (screenshot of StatPlus:macPro license program, AnalystSoft Inc. 2024); Smoothed, the smoothed value at time; Forecast, the predicted value at time; Forecast LCL, forecast lower control limit; Forecast UCL, forecast upper control limit.

**Figure 10:**
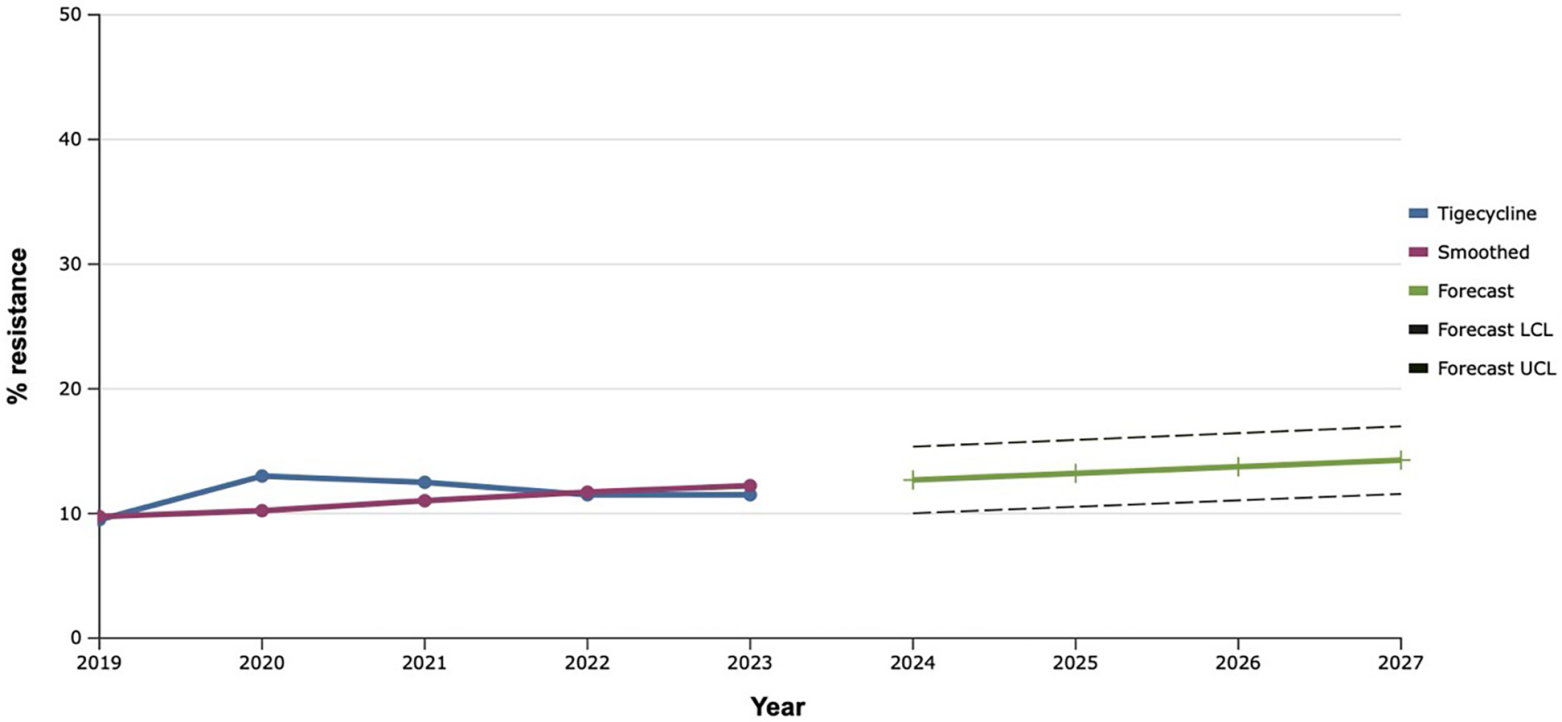
Forecasting the development of S. aureus resistance to linezolid, based on retrospective data from 2019 to 2023 (screenshot of StatPlus:macPro license program, AnalystSoft Inc. 2024); Smoothed, the smoothed value at time; Forecast, the predicted value at time; Forecast LCL, forecast lower control limit; Forecast UCL, forecast upper control limit.

**Figure 11:**
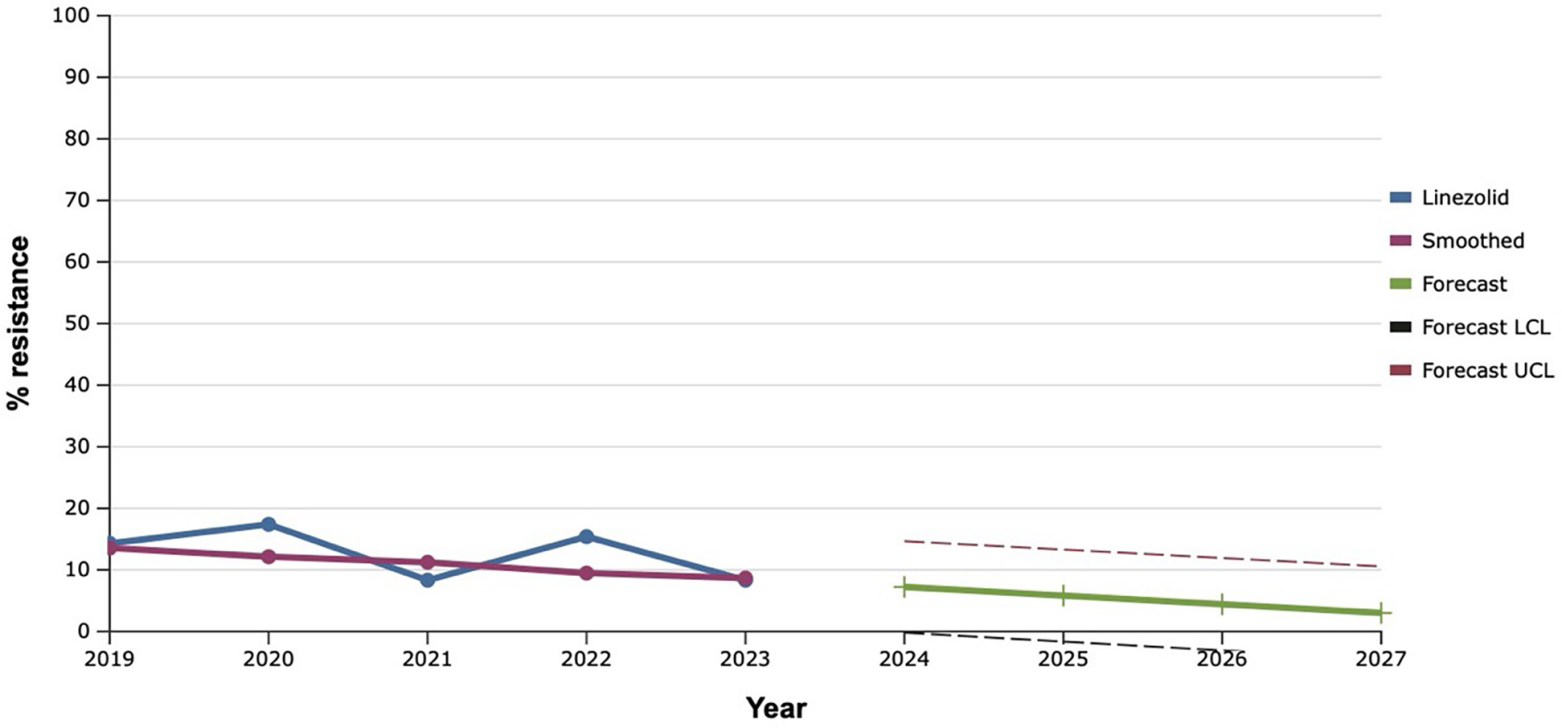
Forecasting the development of S. aureus resistance to tigecycline, based on retrospective data from 2019 to 2023 (screenshot of StatPlus:macPro license program, AnalystSoft Inc. 2024); Smoothed, the smoothed value at time; Forecast, the predicted value at time; Forecast LCL, forecast lower control limit; Forecast UCL, forecast upper control limit.

Among all the antibiotics tested, vancomycin, linezolid, and tigecycline showed the best results, as the level of resistance to them over the past five years did not exceed 20.0% (Figures 9–11). However, the detection of 16.7% vancomycin-resistant staphylococci significantly influenced the outcome of further prediction of the development of resistance among *S. aureus*. Thus, there is a mathematical probability of *S. aureus* acquiring vancomycin resistance at the level of 37.6% by 2027. The only antibiotic that did not show any signs of increasing the proportion of resistant *S. aureus* to it, according to the results of the retrospective analysis, was linezolid. The predictive trend for the next five years was close to zero, which confirmed the promising potential of linezolid in the fight against antibiotic-resistant strains of *S. aureus*.

**Figure 12:**
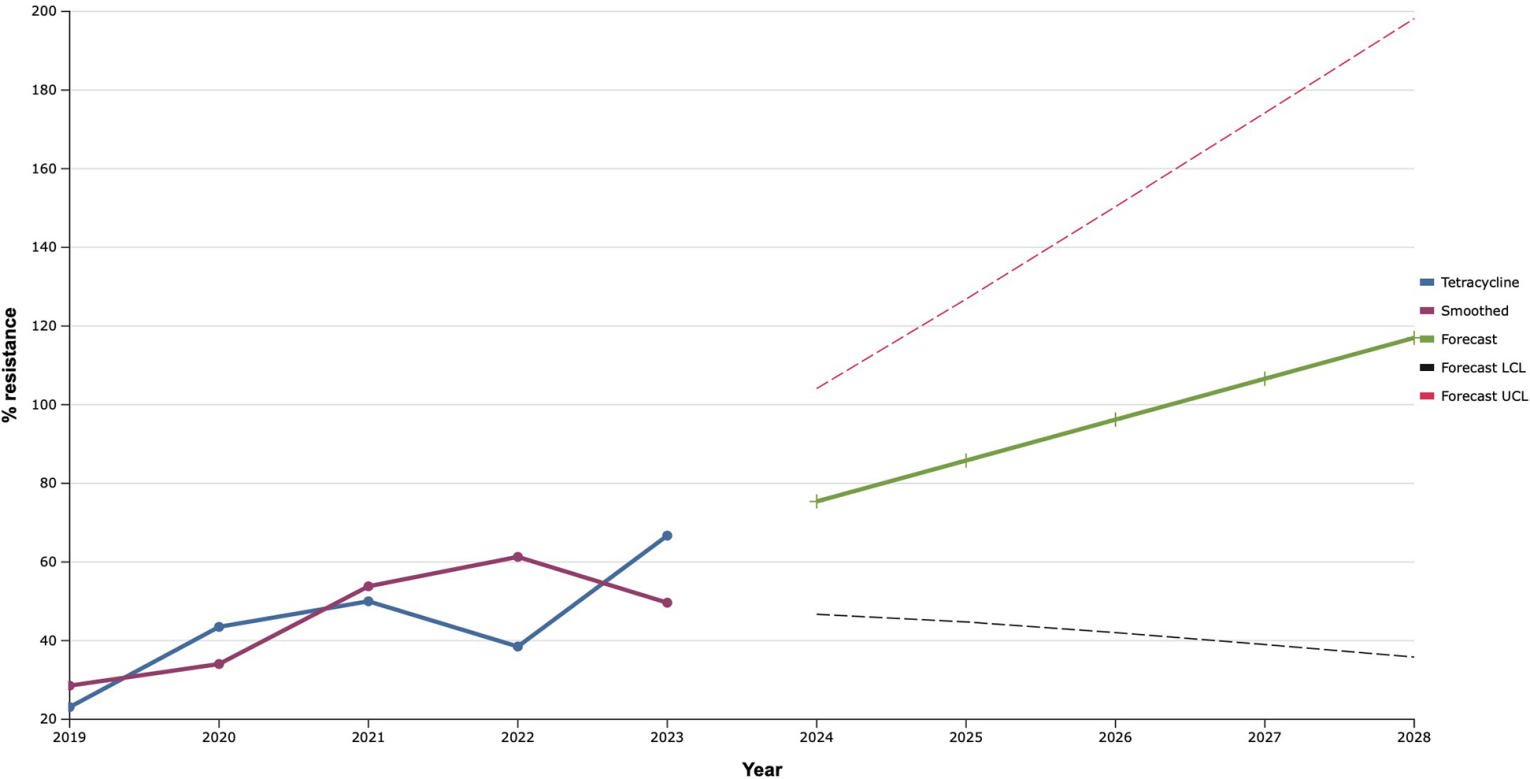
Forecasting the development of S. aureus resistance to tetracycline based on retrospective data from 2019 to 2023 (screenshot of StatPlus:macPro license program, AnalystSoft Inc. 2024); Smoothed, the smoothed value at time; Forecast, the predicted value at time; Forecast LCL, forecast lower control limit; Forecast UCL, forecast upper control limit.

As a result of the study, it was established that the proportion of tetracycline-resistant *S. aureus* isolated from patients with IID of soft-tissue FA increased from 2019 and reached a recorded maximum (66.7%) in 2023 (Figure 12). Based on this, the predicted probability of rapid acquisition of antimicrobial resistance to tetracycline by *S. aureus* strains during the next five years was determined using the exponential smoothing method. Moreover, representatives of this species of bacteria can develop complete (100.0%) resistance to tetracycline as early as 2027.

## 4 Discussion

Today, there are a number of possibilities and methods for forecasting various indicators, both short-term, medium-term, and long-term. This makes it possible to have an idea of the likely course of certain processes in the future, from several days to several years (19). Therefore, it is obvious that the correct choice of forecasting method should be selected in accordance with the needs of the study, the characteristics of the variational data series with the possibility of obtaining the minimum error rate (18). A time series forecasting method that is often used is the exponential smoothing method, which we chose in this study (18, 20). For such forecasts, a set of retrospective values is used to calculate components in the future step by step and are widely used in science, engineering, telecommunications, finance, and economics. Exponential smoothing methods have several advantages over other methods, namely: power, simplicity, and the small number of variables required (19, 21). Among these exponential smoothing methods, the three most commonly used are: Holt's linear method single exponential smoothing, and double or triple exponential smoothing. While the single exponential method is known as simple, Holt's linear method provides trend data forecasts with no seasonality and is of the highest quality (19, 22).

The rapid acquisition of antimicrobial resistance by strains of *S. aureus* is of great concern to scientists and doctors. After all, the level of resistance of representatives of this species to penicillins in China and UK during the last 10 years varied in the range of 17%–33% (23–25). The results obtained by us in Ukraine slightly exceeded these indicators. Along with this, the data of American scientists indicate the development of resistance of *S.aureus* to cefoxitin in 62.8% of cases, which is 19.3% higher than the maximum indicator obtained by us in 2020 (26). It is worth noting that the peaks of resistance to benzylpenicillin and cefoxitin coincided with the beginning of the COVID-19 pandemic and the war in the country, what were probably related. In general, this is obvious, since the frequency of antibiotic prescriptions for the treatment of complicated pneumonias increased significantly during the epidemic. In addition, the fact of increasing antimicrobial resistance during wartime is confirmed not only by the experience of Ukraine (16, 27–29). Of particular concern is the fact that we predict the level of resistance of *S. aureus* strains to β-lactams at the level of 32%–64%. Moreover, according to the recommendations of EUCAST, it is possible to make conclusions about the sensitivity of staphylococci to all β-lactam antibiotics based on sensitivity to benzylpenicillin and cefrxitin. This may indirectly indicate such a level of development of methicillin resistance. Because studies have proven that testing of *S. aureus* for cefoxitin is an experimentally confirmed marker of methicillin resistance (30).

For more than 30 years, scientists around the world have been reporting the acquisition of resistance to fluoroquinolones by *S. aureus* isolates. Moreover, almost a quarter of these are polyresistant species, especially in combination with methicillin resistance (31). The main reasons for such a rapid acquisition of resistance among gram-positive cocci are modification of fluoroquinolone targets, inhibition of permeability due to changes in the outer membrane, overexpression of efflux pumps, and plasmid-mediated resistance. However, more attention has recently been paid to the study of genetic determinants of resistance of staphylococci to quinolone antibiotics, gene mutations and changes in gene regulation. That is why we observed an almost annual increase in the parts of *S. aureus* resistant to fluoroquinolones with a natural predicted increase in the future (32). The results we obtained over the past five years demonstrated a similar global trend. Thus, according to research results in Nigeria, *S. aureus* develop resistance to fluoroquinolones in 31%–33.0% of cases, in Saudi Arabia—33.8%, which fully correlates with similar indicators in Ukraine (33, 34).

The disappointing prognosis for the resistance of *S. aureus* to aminoglycosides, in particular gentamicin, can probably be explained by their widespread use in clinical practice. It is worth noting that the most common mechanism of development of bacterial resistance to aminoglycosides is the synthesis of transferase enzymes, which, by modifying the drug, reduce its effect. However, from the point of view of *S. aureus* as a film-forming bacteria, more attention should be paid to another mechanism of resistance, such as a decrease in enzymatic activity in low-oxygen metabolism during its living in biofilms (35, 36).

It is worth noting that the best results, both retrospectively and prospectively, regarding the development of resistance among *S. aureus* were demonstrated by vancomycin, linezolid, and tigecycline. A similar trend was observed in Latin America, where researchers found almost 99.0% susceptibility to these antibiotics among multidrug-resistant *S. aureus (*37).

Recent research by French scientists through detailed sequencing has determined that isolates of *S. aureus* have a much larger range of genetic determinants responsible for resistance to β-lactams, fluoroquinolones and macrolides, compared to antibiotics of other groups (38). There, it is possible to explain the similarly stable trend of increasing resistance to them in the temporal aspect.

The challenges that have befallen the health care system of Ukraine in recent years significantly affect the spectrum of pathogens of the most common infections and their pattern of antimicrobial resistance (28, 39–41). Therefore, it is important to take into account these changes and develop adequate methods of responding to the perspectives that are expected to develop in the near future.

## 5 Conclusions

The exponential smoothing method clearly outlined the trend of an 18.0% increase in the proportion of *S. aureus* resistant to benzylpenicillin over the next five years. Fluoroquinolone resistance is established based on the result of the sensitivity of staphylococci to norfloxacin, what predicts an increase of the last one among *S. aureus* in the next five years within the range of 20.0%. Mathematical forecasting indicates a potential increase in the level of resistance to aminoglycosides and macrolides among isolates of *S. aureus* in the next five years by 22.7%–33.3% and 35.0% respectively. *S. aureus* can develop complete (100.0%) resistance to tetracycline as early as 2027. There is a mathematical probability of *S. aureus* acquiring vancomycin resistance at the level of 37.6% by 2027. The only antibiotic that did not show any signs of increasing the proportion of resistant *S. aureus* to it, according to the results of the retrospective analysis, was linezolid.

## Data Availability

The raw data supporting the conclusions of this article will be made available by the authors, without undue reservation.
