# Supplementary material for: Forecasting the development of antimicrobial resistance of S. aureus
Source: Front Oral Health. 2025 Jan 9;5:1514070. doi: 10.3389/froh.2024.1514070 (PMC11754302; doi:10.3389/froh.2024.1514070)
Supplement: Supplementary file 1 [file Table1.docx]

Supplementary Table 1

Susceptibility card contents (AST-GP67 for Vitek 2 Systems)

| Antibiotic | FDA indicators for use |
| --- | --- |
| Ampicillin | *Enterococcus* spp., *S. agalactiae* |
| Benzylpenicillin | *Enterococcus* spp., *Staphylococcus* spp. |
| Cefoxitin | *Staphylococcus* spp. |
| Ciprofloxacin | *Enterococcus* spp., *Staphylococcus* spp. |
| Clindamycin | *Staphylococcus* spp. |
| Erythromycin | *Enterococcus* spp., *Staphylococcus* spp. |
| Gentamicin | *Staphylococcus* spp. |
| Linezolid | S. agalactiae, E. faecalis, E. faecium,  S. aureus, S. epidermidis,  S. haemolyticus  *S. agalactiae, E. faecalis, E. faecium, S. aureus, S. epidermidis, S. haemolyticus* |
| Moxifloxacin | MSSA |
| Oxacillin | *Staphylococcus* spp. |
| Rifampicin | *Staphylococcus* spp. |
| Tetracycline | *Enterococcus* spp., *Staphylococcus* spp. |
| Tigecycline | *S. agalactiae, E. faecalis, E. faecium, S. aureus, S. epidermidis, S. haemolyticus, E. casseliflavus* |
| Vancomycin | *Enterococcus* spp., *Staphylococcus* spp. |

Note: FDA - The U.S. Food and Drug Administration; MSSA - Meticillin-Sensitive *S. aureus*
